# Burden among Family Caregivers of Dementia in the Oldest-Old: An Exploratory Study

**DOI:** 10.3389/fmed.2017.00205

**Published:** 2017-11-22

**Authors:** Khin Khin Win, Mei Sian Chong, Noorhazlina Ali, Mark Chan, Wee Shiong Lim

**Affiliations:** ^1^Department of Geriatric Medicine, Tan Tock Seng Hospital (TTSH), Singapore, Singapore; ^2^Institute of Geriatrics and Active Ageing, Tan Tock Seng Hospital (TTSH), Singapore, Singapore; ^3^Geriatric Education and Research Institute (GERI), Singapore, Singapore

**Keywords:** dementia, oldest-old, caregiver burden, Zarit Burden Interview, dimensions

## Abstract

**Background:**

With >85 years, the fastest growing age segment in developed countries, dementia in the oldest-old is projected to increase exponentially. Being older, caregivers of dementia in oldest-old (CDOO) may experience unique challenges compared with younger-age groups. Thus, we aim to explore demographic characteristics and burden pattern among CDOO.

**Methods:**

We studied 458 family caregiver-patient dyads attending an outpatient memory clinic. We classified patients into three age-groups: <75, 75–84, and ≥85 years. We measured caregiver burden using the Zarit Burden Interview (ZBI) 4-factor structure described by Cheah et al. (1). We compared care recipient characteristics, caregiver demographics, and ZBI total/factors scores between the three age-groups, and performed 2-way analysis of variance (ANOVA) to ascertain the effect of age-group by disease severity interaction.

**Results:**

Oldest-old care recipients were more impaired in cognitive function and instrumental ADL; there was no difference in behavior and basic ADL. Compared with the other two age-groups, CDOO were older (mean age: 50.4 vs 55.5 vs 56.8 years, *P* < 0.01), and overwhelmingly adult children (85.9%) as opposed to spouses (5.3%). CDOO also had higher ZBI total score, role strain, and personal strain (all *P* < 0.05). However, there was no difference in worry about performance scores. 2-way ANOVA did not reveal significant age-group by disease severity interaction for ZBI total and factor scores, although distinctive differences were seen between role/personal strain with worry about performance in mild cognitive impairment and very mild dementia.

**Conclusion:**

Our study highlighted that CDOO were mainly older adult children who experienced significant role and personal strain independent of disease severity while caring for their family member with more impaired cognitive and physical function. These results pave the way for targeted interventions to address the unique burden faced by this rapidly growing group of caregivers.

## Introduction

Globally, the oldest-old population, variously defined as 80 or 85 years and older, has emerged as the fastest growing age segment, especially in developed countries. The oldest-old population is projected to increase 151 percent between 2005 and 2030, far outstripping the 21 percent increase for those under age 65 and 104 percent increase for those aged 54 years and above (2). In line with this worldwide trend, the prevalence of the oldest-old population in Singapore has grown exponentially from 4,500 in 1980 to over 27,800 in 2009 (3).

The prevalence of age-associated diseases such as dementia is expected to mirror this worrying global demographic trend of population aging, such that growth of dementia in the oldest-old (DOO) is expected to exhibit a corresponding exponential rise that far outstrips other age groups. Recent studies support this assertion that the oldest-old represents the fastest growing population with dementia. The WiSE study (4) conducted in Singapore in 2013 showed that the prevalence of dementia was 10% in the elderly population above 60 years of age and the likelihood of dementia for those 85 years and above were 18.4 times higher compared to those aged 60–74 years. A systematic review and metaanalysis on the global prevalence of dementia reported that 18.7% of those between 85 and 89 years and 35.4% of those above 90 years of age in the South East Asian regions were estimated to be affected by dementia (5).

This increase in DOO coincides with a dramatic decline in the potential support ratio, namely persons aged 20–64 per person aged 65 or older. Population projections for Singapore predict that the potential support ratio will drop from 5.7 in 2015 to around 2.1 by 2030, with similar declines expected in most countries worldwide (6). Because the growth in health-care professionals trained in dementia care is unlikely to keep pace with this burgeoning demand, it is anticipated that the responsibility of caring for persons with DOO will increasingly fall upon informal family caregivers such as spouses, children, grandchildren, siblings, or other relatives. As family is expected to be the primary source of care going forward, especially in Asian populations, understanding the potential challenges faced by family caregivers of dementia in oldest-old (CDOO) is, therefore, of great salience and importance.

Caregivers of dementia in oldest-old are expected to be older in age and are likely burdened with more concerns such as health issues, family commitments, or financial constraint compared to their younger counterparts. Furthermore, persons with DOO are likely to require higher care needs, such that the caregiving role can have deleterious impact on one’s physical and psychological well-being. Despite this, the majority of research in caregivers in dementia focuses on the younger old, and there is limited literature that specifically pertains to CDOO and the caregiving burden that they may experience relative to the younger-old age group. For instance, the study by Liu et al. among Chinese adult children taking care of their oldest-old parents was limited to care recipients who were relatively cognitively well and did not require much assistance in their activities of daily living (7). More recently, Liu et al. reported that Chinese adult children experience strain from worry about performance when providing care for their oldest-old parents (8). These results suggest that CDOO may face unique challenges in their caregiving role, particularly in Asian populations that are often heavily influenced by notions of filial piety and obligatory care (9, 10).

In light of this, it is imperative that the study of caregiver strain in DOO is approached from a multidimensional perspective as opposed to solely assessing the total burden score, constituting what is effectively a unidimensional approach. Caregivers with an identical score may express difference aspects of burden (11); while one may be affected by physical demands of care recipients, other may be worried about his caregiving performance (9). Recent studies suggest that the different dimensions of caregiver burden as measured by the Zarit Burden Interview (ZBI) among Chinese informal caregivers for dementia exhibit different trajectories across the severity of dementia (12). Thus, using the validated 4-facture structure proposed by Cheah et al. (1), we aim to describe care recipient and caregiver characteristics, as well as caregiver burden, in DOO compared to young-old (below 75 years old) and middle-old (75–84 years old) individuals with dementia. Our secondary objective is to compare the burden pattern across the spectrum of disease severity among the three age-groups.

## Materials and Methods

### Study Design and Participants

This is a cross sectional study involving 458 caregiver-patient dyads of community dwelling older adults who were referred to the Memory Clinic, Tan Tock Seng Hospital, Singapore from January 2010 to December 2011. The Memory Clinic is a tertiary referral clinic within the Department of Geriatric Medicine that receives referrals from polyclinics, family physicians, other restructured hospitals, and other departments within Tan Tock Seng Hospital. Patients are referred for assessment of cognitive and memory difficulties as well as behavioral issues without significant functional limitations. The annual attendance at the Memory clinic in 2010 and 2011 were 500 and 943 new cases, respectively.

Our inclusion criteria were: (1) patients aged 55 years and older with a Clinical Dementia Rating (CDR) global score of >0 and with a diagnosis of mild cognitive impairment (MCI) or dementia (13); (2) presence of a primary caregiver, defined as the family member who was most involved in the provision of daily care and familiar with the patient’s social and medical status; (3) completion of the 22-item ZBI questionnaire. We excluded caregivers who were non-family members (for example, domestic helpers or friends), unable to understand the Chinese or English language, or unable to complete the ZBI questionnaire. The study was approved by the Institutional Review Board of the National Healthcare Group. As this study involved the retrospective review of medical records of patients attending the Memory clinic as part of a registered database (TTSH/2008-0027), waiver of informed consent was approved by the Institutional Review Board of the National Healthcare Group.

### Assessment

All participants underwent standardized assessment by a geriatrician and nurse clinician, blood investigations, neuroimaging and whenever relevant, psychometric assessment. A consensus meeting was conducted to determine the diagnosis, etiology, and staging of cognitive impairment based upon multi-disciplinary inputs from the physician, nurse clinicians, and psychologist. Dementia was diagnosed based on the *Diagnostic and Statistical Manual of Mental Disorders, Fourth Edition* (DSM-IV) criteria, and etiology classified using published international criteria for dementia, as previously described (11). MCI was diagnosed using the revised Petersen criteria (14). The severity of cognitive impairment was rated using the locally validated clinical dementia rating scale (CDR) (15). CDR 0 indicates no cognitive impairment; CDR 0.5 designates either MCI or very mild dementia; and CDRs 1, 2, and 3 indicate mild, moderate, and severe dementia, respectively (16).

### Measurements and Instruments

We collected baseline demographic data of care recipients and their caregivers, including age, gender, ethnicity, and educational level. We also collected information on caregiver characteristics such as relationship and co-residence with care recipients.

We assessed cognitive performance using the locally validated Chinese Mini-Mental State Examination (total score of 28) (17). Functional status was assessed using the modified Barthel Index (score 0–100) (18) and Lawton instrumental activities of daily living (IADL) (score 0–23) (19). Neuropsychiatric symptoms were assessed using the Neuropsychiatric Inventory Questionnaire (NPI-Q); severity (20) and carer distress scores (21) were computed separately. These assessments form part of the routine clinical evaluation for all patients attending the Memory Clinic and are routinely gathered and documented in the files.

Caregiver burden was assessed using the 22-item ZBI questionnaire, which was administered either in English or Chinese. Each item was scored on a 5-point Likert scale, ranging from 0 = “never” to 4 = “nearly always,” yielding a total score ranging from 0 to 88. We used the 4-factor structure reported by Cheah et al., which accounted for 62.2% of the variance with good internal consistency (1): (1) factor 1: role strain from demands of care and social impact on caregiver (40.6% of variance); (2) factor 2: role strain from lack of confidence or control over the situation (9.7% of variance); (3) factor 3: personal strain due to psychological impact on caregiver (6.4% of variance); and (4) factor 4: worry about caregiving performance (5.6% of variance).

### Statistical Analysis

We performed descriptive and analytical statistics using SPSS (version 21.0; SPSS Inc., Chicago, IL, USA). All tests were 2-sided and the level of significance set at 0.05. We categorized caregiver-patient dyads into three groups based upon the age of the care recipient: young-old (aged below 75 years), middle-old (aged between 75 and 84 years), and oldest-old (aged 85 years and above). We compared the characteristics of care recipients and caregivers between the three age groups, as well as ZBI total and factor scores stratified by relationship with care recipient. We conducted *X*^2^ test for categorical variables and one-way analysis of variance (ANOVA) with *post hoc* comparison corrected for the Turkey HSD test was used for continuous variables. We further performed two-way ANOVA to ascertain the effect of age group by disease severity interaction on caregiver burden (ZBI total and individual factor scores).

## Results

### Characteristics of Caregiver-Patient Dyads

Our final sample of 458 caregiver-patient dyads was predominantly of Chinese ethnicity (Table 1). The mean age of care recipients was 76.5 years (SD 7.4 years) with 59% of female gender. Alzheimer’s dementia was the major etiology (46.7%), followed by other dementias (i.e., not vascular dementia nor mixed dementia) (18.6%), vascular dementia (17%), and mixed dementia (6.1%). About half of the recipients were rated CDR 1 (44.3%) followed by CDR 2 (27.7%), CDR 0.5 dementia (12.7%), and MCI (12%) and CDR 3 (3.3%).

**Table 1:** Characteristics of caregiver and care recipient dyads.

	Care recipients	Caregivers
	*n* = 458	*n* = 458
**Demographics**		
Age	76.5 ± 7.4	53.8 ± 13.5
Female gender, *n* (%)	270 (59)	287 (62.7)
Ethnicity, *n* (%)		
Chinese	409 (89.3)	
Malay	29 (6.3)	
Indian	14 (3.1)	
Others	6 (1.3)	
Years of formal education	4.8 ± 4.6	11 ± 4.5
Relationship with care recipients, *n* (%)		
Spouse		139 (30.3)
Adult children		295 (64.5)
Others		20 (4.4)
Living with care recipient, *n* (%)		351 (76.6)
**Disease characteristics**		
Dementia type, *n* (%)		
Alzheimer’s dementia	214 (46.7)	
Vascular dementia	78 (17)	
Mixed dementia	28 (6.1)	
Others	85 (18.6)	
Global CDR score, *n* (%)		
CDR 0.5 (mild cognitive impairment)	55 (12)	
CDR 0.5 (very mild dementia)	58 (12.7)	
CDR 1 (mild dementia)	203 (44.3)	
CDR 2 (moderate dementia)	127 (27.7)	
CDR 3 (severe dementia)	15 (3.3)	
CMMSE (range 0–28)	16.6 ± 6	
BADL (range 0–100)	92.8 ± 36.7	
IADL (range 0–23)	12.1 ± 5.9	
Behavioral symptoms		
NPI-Q severity (range 0–36)	5.6 ± 5	
NPI-Q distress (range 0–60)		5.8 ± 7.2
**Caregiver burden—ZBI scores**		
Total ZBI (range 0–88)	25.0 ± 17.4	
Factor 1 (range 0–36)	12.1 ± 8	
Factor 2 (range 0–20)	3.7 ± 4.3	
Factor 3 (range 0–24)	6.2 ± 5.3	
Factor 4 (range 0–8)	3.1 ± 2.4	

*Mean (SD) unless otherwise stated*.

*BADL, Barthel index of Basic Activities of Daily Living; CDR, clinical dementia rating; CMMSE, Chinese Mini Mental Status Examination; IADL, Instrumental Activities of Daily Living; NPI-Q, Neuropsychiatric Inventory Questionnaire; ZBI, Zarit Burden Interview*.

The mean age of caregivers was 53.6 years (SD 13.5 years) and majority were daughters (42.4%), followed by spouses (30.3%) and sons (22.1%). The majority of caregivers (76.6%) resided with the care recipient. The NPI-Q severity and distress mean scores were 5.6 (SD 5), and 5.8 (SD 7.2), respectively. The mean ZBI scores are: total ZBI, 25 (SD 17.4); factor 1, 12.1 (SD 8); factor 2, 3.7 (SD 4.3); factor 3, 6.2 (SD 5.3); and factor 4, 3.1 (SD 2.4), respectively.

### Care Recipient Characteristics

There was no difference in gender or ethnicity across the age groups (Table 2). Compared with the other two age-groups, care recipients with DOO are older (*P* < 0.01), more likely to be female (*P* < 0.05), and have lower educational level (*P* < 0.01). Alzheimer’s disease was the predominant etiologic diagnosis for all three groups. Care recipients in the young-old groups was more likely to present at earlier stages such as MCI (18.1%) or CDR 0.5–1 dementia (60%); in contrast, close to half (47.4%) of DOO patients presented with CDR 2–3 moderate-to-severe dementia. DOO patients also scored lower on the CMMSE (*P* = 0.003) and were more impaired in IADL (*P* < 0.01) although less impaired in BADL. Though the NPI-Q severity score was similar across the three age groups, NPI-Q carer distress score was higher in the DOO group.

**Table 2:** Comparison of care recipient and disease characteristics.

	Young-old	Middle-old	Oldest-old	*P-*value
		
	<75 years	75–84 years	≥85 years	
		
	*n* = 155	*n* = 246	*n* = 57	
Age of care recipients	68.5 ± 5	78.9 ± 2.7^a^	88 ± 2.7^b,c^	<0.01
Female gender, *n* (%)	90 (58.1)	136 (55.3)	44 (77.2)	0.01
Ethnicity, *n* (%)				
Chinese	137 (88.4)	221 (89.8)	51 (89.5)	0.56
Malay	13 (8.4)	15 (6.1)	1 (1.8)	
Indian	4 (2.6)	5 (2)	5 (8.8)	
Others	1 (0.6)	5 (2)	0 (0)	
Years of formal education	5.8 ± 4.7	4.6 ± 4.7^a^	3.4 ± 4.6^b^	<0.01
Primary diagnosis, *n* (%)				0.626
Alzheimer’s dementia	66 (51.6)	121 (53.8)	27 (51.9)	
Vascular dementia	23 (18)	42 (18.7)	13 (25)	
Mixed dementia	13 (10.2)	13 (5.8)	2 (3.8)	
Others	26 (20.3)	49 (21.8)	10 (19.2)	
Global CDR score	2.7 ± 1.1	3.1 ± 0.9^a^	3.3 ± 1.0^b^	<0.001
Clinical staging, *n* (%)				0.004
Mild cognitive impairment	28 (18.1)	22 (8.9)	5 (8.8)	
Very mild dementia	25 (16.1)	27 (11)	6 (10.5)	
Mild dementia	68 (43.9)	116 (47.2)	19 (33.3)	
Moderate dementia	29 (18.7)	75 (30.5)	23 (40.4)	
Severe dementia	5 (3.2)	6 (2.4)	4 (7)	
CDR sum of boxes	5.4 ± 3.9	6.4 ± 3.8^a^	8.03 ± 4.7^b,c^	<0.01
Cognitive status				
CMMSE (0–28)	17.8 ± 6.3	16.1 ± 5.7^a^	15 ± 6.4^b^	0.003
Functional status				
BADL (0–100)	93.6 ± 13.6	91.2 ± 16.3	97.7 ± 96.3	0.461
IADL (0–23)	13.9 ± 5.8	11.7 ± 5.6^a^	8.7 ± 5.4^b,c^	<0.001
Behavioral symptoms				
NPI-Q severity score (0–36)	5.6 ± 5.1	5.5 ± 4.9	5.6 ± 5.3	0.943
NPI-Q carer distress score (0–60)	5.7 ± 7	5.6 ± 3	6.7 ± 8.7	0.78

*Values are reported as mean ± SD or frequency (%) unless otherwise stated*.

*Post hoc comparison with Turkey HSD test (*P* < 0.05)*.

*^a^Young-old vs middle-old*.

*^b^Young-old vs oldest-old*.

*^c^Middle-old vs oldest-old*.

*BADL, Barthel index of Basic Activities of Daily Living; CDR, Clinical Dementia Rating; CMMSE, Chinese Mini Mental Status Examination; IADL, Instrumental Activities of Daily Living; NPI-Q, Neuropsychiatric Inventory Questionnaire*.

### Caregiver Characteristics and Burden

Compared with the younger-old age groups, CDOO were older in age (*P* < 0.01), and were mainly adult children (daughters followed by the sons) or others, compared with spouses and daughters in the younger-old age groups (Tables 3 and 4). When caregiver age was stratified by relationship, adult–child CDOO were older compared with the other two age groups. Spousal CDOO also tended to be older compared with the young-old age group (72.0 vs 64.6 years), although the converse was true for non-spousal non-children CDOO (58.0 vs 47.6 years).

**Table 3:** Comparison of caregiver characteristics.

	Young-old	Middle-old	Oldest-old	*P-*value
		
	<75 years	75–84 years	≥85 years	
		
	*N* = 155	*n* = 246	*n* = 57	
Age, overall group	50.4 ± 14.3	55.2 ± 13.4^a^	56.8 ± 10^b^	<0.01
Spouse	64.6 ± 7.1	72.9 ± 8.1^a^	72.0 ± 9.6	<0.01
Daughters	40.0 ± 7.5	48.9 ± 5.5^a^	56.5 ± 7.1^b,c^	<0.01
Sons	39.4 ± 8	48.3 ± 6.5^a^	57.5 ± 6.2^b,c^	<0.01
Others	58.0 ± 0	46.6 ± 21.4	47.6 ± 20	0.783
Female gender, *n* (%)	96 (64.4)	152 (67.9)	39 (69.6)	0.707
Relationship with care recipient, *n* (%)				<0.01
Spouse	65 (42.8)	71 (29)	3 (5.3)	
Daughters	55 (36.2)	105 (42.9)	34 (59.6)	
Sons	30 (19.7)	56 (22.9)	15 (26.3)	
Others	2 (1.3)	13 (5.3)	5 (8.8)	
Living with care recipient, *n* (%)	129 (86)	181 (79.7)	41 (73.2)	0.087
Years of formal education	11.1 ± 4.8	10.8 ± 4.5	11.2 ± 4.2	0.889

*Values are reported as mean ± SD or frequency (%) unless otherwise stated*.

*Post hoc comparison with Turkey HSD test (*P* < 0.05)*.

*^a^Young-old vs middle-old*.

*^b^Young-old vs oldest-old*.

*^c^Middle-old vs oldest-old*.

**Table 4:** Comparison of Zarit Burden Interview (ZBI) total and factor scores across the three age groups stratified by relationship.

All subjects	Young-old	Middle-old	Oldest-old	*P-*value
	
	<75 years	75–84 years	≥85 years	
	
	*n* = 155	*n* = 246	*N* = 57	
Total ZBI (range 0–88)	22.9 ± 16.8	25.1 ± 17.1	30.5 ± 19.2^a^	0.017
Factor 1 (0–36)	11 ± 7.9	12.2 ± 7.9	14.3 ± 8.3^a^	0.025
Factor 2 (0–20)	3.2 ± 3.9	3.7 ± 4.2	5.2 ± 5.1^a,b^	0.008
Factor 3 (0–24)	5.7 ± 5.1	6.1 ± 5.2	7.9 ± 5.8^a,b^	0.018
Factor 4 (0–8)	3 ± 2.5	3.2 ± 2.3	3.1 ± 2.4	0.876

**Spouses**	*N* = 65	*n* = 71	*n* = 3	

Total ZBI (range 0–88)	20.8 ± 16.3	19.7 ± 18.2	35 ± 20.3	0.324
Factor 1 (0–36)	10.6 ± 8	10.2 ± 8.3	17.3 ± 8.5	0.328
Factor 2 (0–20)	2.4 ± 3	2.8 ± 4.1	5.6 ± 6	0.319
Factor 3 (0–24)	5.1 ± 5	4.5 ± 5.3	9.3 ± 5.7	0.268
Factor 4 (0–8)	2.6 ± 2.9	2.2 ± 2.5	2.6 ± 2.3	0.576

**Daughters**	*N* = 55	*n* = 105	*n* = 34	

Total ZBI (range 0–88)	24.5 ± 16	27.9 ± 16.3	30.1 ± 19	0.277
Factor 1 (0–36)	11.8 ± 7.9	13.3 ± 7.5	14.3 ± 8.3	0.276
Factor 2 (0–20)	3.3 ± 3.5	4.1 ± 4.2	5.3 ± 5.1	0.092
Factor 3 (0–24)	5.9 ± 5.1	6.8 ± 5.1	7.5 ± 5.9	0.356
Factor 4 (0–8)	3.6 ± 2.3	3.7 ± 2.2	3.1 ± 2.3	0.379

**Sons**	*N* = 30	*n* = 56	*n* = 15	

Total ZBI (range 0–88)	24.7 ± 17.4	27.01 ± 16.1	31.8 ± 21	0.429
Factor 1 (0–36)	10.6 ± 7.2	12.8 ± 7.7	14.5 ± 9	0.252
Factor 2 (0–20)	4.4 ± 5	4 ± 4.2	5.5 ± 5.8	0.557
Factor 3 (0–24)	6.4 ± 4.9	6.6 ± 4.8	9.1 ± 6.1	0.204
Factor 4 (0–8)	3.3 ± 2.0	3.7 ± 2.0	2.8 ± 2.5	0.339

*Post hoc comparison with Turkey HSD test (*P* < 0.05)*.

*^a^Young-old vs oldest-old*.

*^b^Middle-old vs oldest-old*.

In addition, CDOO expressed higher caregiver stress with higher total ZBI, role strain/demands, role strain/control, and personal strain (all *P*-value *P* < 0.05). In contrast, there was no difference in worry about performance across the three groups. When stratified by relationship, spousal CDOO endorsed higher ZBI total score and all factor scores with the exception of worry about performance; however, these results were not statistically significant, possibly due to small numbers (*N* = 3) in the spousal CDOO group. For adult–child CDOO, there is also a trend for higher total, role strain, and person strain scores, albeit not statistically significant. In contrast, worry about performance was lowest in CDOO compared with the other two age groups. Adult-son CDOO also showed higher factor three scores (psychological impact from caregiving) than adult-daughter CDOO though this difference was not statistically significant by independent sample *t*-test.

### Effect of Disease Severity on Caregiver Burden

**Figure 1:**
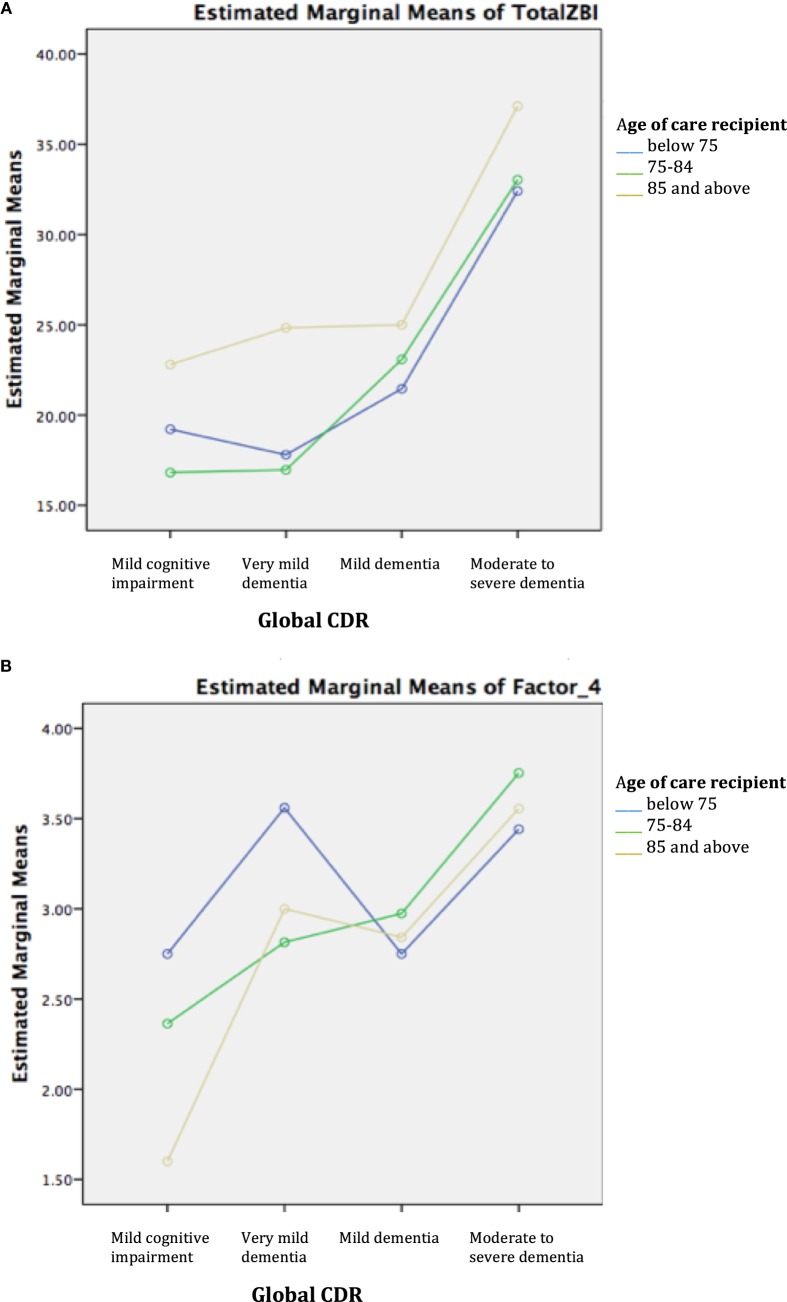
**(A)** Trend of total Zarit Burden Interview (ZBI) across disease severity by age group. **(B)** Trend of factor 4 across disease severity by age group.

Two-way ANOVA revealed that there was no statistically significant interaction between age group and disease severity for ZBI total and factor scores. Examination of the graphical plots yielded interesting insights about how the trend of burden scores across disease severity for DOO differs from the younger-old age groups. For instance, ZBI total score was highly endorsed among CDOO in MCI and very mild dementia and progressively increased with disease severity to merge with the curves for the other age groups (Figure 1A). A comparable trend was noted for factors 1–3. In contrast, for factor 4, a reverse pattern was noted with CDOO endorsing the lowest score in the MCI stage (Figure 1B). Factor 4 scores subsequently increased with dementia severity to merge with the curves for the other two groups.

## Discussion

To our knowledge, this is the first study to shed light on the unique challenges faced by caregivers of the oldest-old care recipients with dementia, who tend to present at more severe stages of dementia and are more cognitively and functionally impaired. An earlier Chinese study of oldest-old caregivers was limited to care recipients who were cognitively well and required less assistance in basic and IADL (7, 8). An added strength of our study is the use of a multidimensional approach to illuminate the pattern of caregiver burden by relationship and across the severity of cognitive impairment. Our results reveal that CDOO are older and typically an older adult–child or a younger non-spousal/non-child family member. Compared with their counterparts looking after the middle-old and young-old age groups, CDOO experience higher caregiver burden in the domains of role strain and personal strain but not worry about performance, even in the earliest stages of MCI and very mild dementia.

Our study affirmed the fact that relative to caregivers looking after the younger-old with dementia, CDOO experienced greater overall burden, increased demands, and lack of control over situation leading to role strain, and the psychological impact of personal strain. Notably, CDOO endorsed higher stress from behavioral symptoms even with comparable severity of behavioral symptoms, alluding to how the strain of caregiving may have affected their appraisal of the stress arising from behavioral symptoms. As individuals with dementia survive into the oldest-old age group, the fewer the number of spouses who remain as the primary caregiver and the more likely that older adult children take over this role. In a predominantly Chinese Asian society such as ours, adult–child CDOO may be thrusted into the caregiving role as there are social expectations to care for elderly family members, and filial piety is a core value in Chinese culture. Being generally older and approaching the age of retirement, adult–child CDOO may struggle even more if they have not yet made adequate arrangements for their jobs, their families, and post retirement financial security, or have concomitant health issues. Pearlin et al. reported that two types of role conflicts may appear in adult–child caregivers: one is the conflict between the caregiver role and the roles in their nuclear family, such as spouse and parent; the other one is the conflict between the caregiver role and their roles in the workplace, such as employer or employee (22). Zhan also reported that caregivers who assisted with mostly instrumental care reported greater levels of emotional and relational frustration (23). It is, therefore, not surprising that adult–child CDOO who are often unprepared for their transition into the caregiving role with increased demands of providing assistance in instrumental ADL and physical care and coping with dementia behaviors, experience the resultant emotional and relational strain arising from the caregiving role.

Our study also highlighted the differences in burden pattern among spousal and adult–child CDOO. Both groups endorsed endorsed higher ZBI total score and factor 1–3, although the scores are higher in spousal than adult–child CDOO. This observed trend might be due to factors such as closer relationship of spouses with care recipients (24), co-residence with care recipients, and concomitant health and physical ailments, leading to a greater degree of perceived stress when providing long-term care (25). Comparing between the children relationships, adult-son CDOO endorsed higher factor 3 score compared to their daughter counterparts. This may be attributable to the psychological impact arising from risk of role overload from conflicting responsibilities. Because sons are generally more esteemed than daughters in more traditional Chinese families, adult-son CDOO may be expected to play a leading role in care provision for their aged parents, and this in turn can create psychological strain if they feel sandwiched between this caregiving role on top of their work and family commitments (11, 26).

In addition, our study identified that the higher overall burden among CDOO is accounted for by role and personal strain. Contrary to the findings of Liu et al. (8) that the adult–child caregiver experience significant burden from worry about performance, the adult–child CDOO (both daughters and sons) in our study paradoxically experience lower worry about performance compared with the younger-old age groups. Worry about performance is self-appraisal of their caregiving performance, which encompasses both positive and negative valences (27). On the positive end, caregivers may have positive perceptive of their capability to take good care of their family member with dementia. Conversely, worry about performance may signify negative feelings of inadequacy and self-criticism leading to guilt and shame (9). It is, therefore, important to consider the difference in context between the two studies when interpreting the seemingly discrepant findings. Liu et al. (8) examined elders who were cognitively well and required less assistance in basic and IADL, hence their adult–child caregivers would naturally “worry” how they can take better care of their parents to maintain the overall good health. In contrast, our study involved oldest-old care recipients who present at more advanced stages of dementia with increased physical and emotional care needs. Having to juggle multiple competing stressors such as personal health, family commitments, and financial issues on top of their caregiving role, adult–child CDOO not surprisingly experience role and personal strain while having a lower predilection for worry about performance stress. Our results, therefore, corroborate the findings of Lim et al. that in the context of dementia, younger age is the most important predictor of worry about performance stress even amongst adult–child caregivers (9).

Indeed, caring of frail elderly individuals with dementia can be challenging causing both physical and mental health problems in caregivers, yet, the responsibilities of caring for DOO will still fall upon the informal caregivers. So, it is vital to provide caregiver support interventions to reduce the burden faced by CDOO. Support at the individual level can be beneficial in reducing physical and psychological burden of caregiving. Interventions such as creating network for caregiver support, respite care arrangement, counseling on coping abilities and financial support can reduce caregiving burden and improve caregiving abilities in this vulnerable group of DOO patient–caregiver dyads.

Several limitations are worth highlighting. First, because this is a cross sectional study, reverse causality cannot be excluded. Further longitudinal studies will be required to affirm the findings. Second, our study sample of oldest-old care recipients is relatively small; hence the results of our exploratory study need to be further verified in larger study populations. Third, our study population of patients with milder severity of dementia of predominantly Chinese Asian ethnicity may limit the generalizability of our findings to other socio-cultural context. Fourth, we excluded friends or employed caregivers who may experience different patterns of burden compared to family caregivers. Future studies should examine the impact on psychological well-being by examining outcomes such as depression, anxiety, and quality of life. Finally, we did not collect data on certain variables that can influence the severity of caregiver stress and burden pattern, such as the duration of caregiving and the number of caregivers who are involved in the care.

In summary, our study demonstrated the unique burden faced by the caregivers of the oldest-old with dementia, who were mainly older adult children experiencing significant role and personal strain but not worry about performance from looking after their family members with more impaired cognition and physical function. Although this unique pattern of caregiver burden is generally independent of disease severity, overall burden, role strain, and personal strain are already high in the early stages of cognitive impairment, and increases further as the disease progresses. The results of our exploratory study provide insight, which paves the way to address the unique burden faced by this vulnerable group of caregivers through individualized interventions that target coping resources and stressors to increase caregiving mastery, which acts as a buffer against the deleterious impact of role and personal strain from the caregiving role (28).

## Ethics Statement

The study was approved by the Institutional Review Board of the National Healthcare Group. As this study involved the retrospective review of medical records of patients attending the Memory clinic as part of a registered database (TTSH/2008-0027), waiver of informed consent was approved by the Institutional Review Board of the National Healthcare Group.

## Author Contributions

KW conducted the study, carried out the statistical analysis, and wrote the manuscript. MC, NA, and MC supported the development of study design and methodology, and reviewed the manuscript. WL designed the study and supported the writing of the manuscript.

## Conflict of Interest Statement

The authors declare that the research was conducted in the absence of any commercial or financial relationships that could be construed as a potential conflict of interest.
